# Supplementary material for: MetaRibo-Seq measures translation in microbiomes
Source: Nat Commun. 2020 Jun 29;11:3268. doi: 10.1038/s41467-020-17081-z (PMC7324362; doi:10.1038/s41467-020-17081-z)
Supplement: Supplementary file 10 — Supplementary Data 7 [file 41467_2020_17081_MOESM10_ESM.zip › File2/Confidence_High_Taxonomy/383845_out.krona.html]

Javascript must be enabled to view this page.

members
magnitude
magnitudeUnassigned
count
unassigned
taxon
rank

383845\_out

2

2
superkingdom
2

phylum
201174
2

class
84998
2

84999
order
2

family
84107
2

2
102106
genus

1
2292330
species

SRS143466\_contig\_number\_26465


SRS078419\_contig\_number\_35996
species
74426
1
